# The efficacy and safety of aspirin in preventing venous thrombosis in major orthopedic surgery: An updated meta-analysis of randomized controlled trials

**DOI:** 10.1097/MD.0000000000035602

**Published:** 2023-10-20

**Authors:** Heng-Zhi Liu, Jie Liang, Ai-Xin Hu

**Affiliations:** a Department of Orthopaedics, The First College of Clinical Medical Science, China Three Gorges University, Yichang, China; b Department of Orthopaedics, Yichang Central People’s Hospital, Yichang, China.

**Keywords:** anticoagulants, aspirin, major orthopedic surgery, proximal deep vein thrombosis, pulmonary embolism

## Abstract

**Background:**

Major orthopedic surgery, including hip and knee replacement and lower extremity trauma fractures surgery, is associated with a high risk of venous thromboembolism (VTE), especially proximal deep vein thrombosis (DVT), and pulmonary embolism (PE), and is linked with high morbidity and mortality rates. Chemical anticoagulation is routinely used to prevent VTE, with previous meta-analyses reporting on the efficacy and safety of aspirin and other anticoagulants, however, opinions are divided. In the past 2 years, several large randomized controlled trials have been published, therefore, we reanalyzed aspirin efficacy and safety when compared with other anticoagulants in preventing VTE in major orthopedic surgery.

**Methods:**

Using PubMed, The Cochrane Library, Embase, and Web of Science databases, we conducted a RCT search in August 2023. The main outcomes included VTE, proximal DVT or PE. Additional outcomes included bleeding events, wound complications, wound infections, blood transfusions, and death events.

**Results:**

In total, 17 eligible articles, involving 29,522 patients (15,253 aspirin vs 14,269 other anticoagulant cases), were included. Primary outcomes showed that VTE incidence was more high in the aspirin group when compared with other anticoagulants (risk ratio [RR] = 1.45, 95% confidence interval [CI] = 1.18–1.77, *P* = .0004) and proximal in the aspirin group the DVT and/or PE incidence was significantly higher in the aspirin group when compared with other anticoagulants (RR = 1.19, 95% CI = 1.02–1.39, *P* = .03). No significant secondary outcome differences were identified in the aspirin group when compared with other anticoagulants (bleeding events [RR] = 0.83, 95% CI = 0.63–1.10, *P* = .20); wound complications (RR = 0.45, 95% CI = 0.20–1.04, *P* = .06); wound infection (RR = 1.08, 95% CI = 0.85–1.38, *P* = .53); blood transfusion events (RR = 1.00, 95% CI = 0.84–1.19, *P* = 1.00) and death events (RR = 1.11, 95% CI = 0.78–1.57, *P* = .55).

**Conclusions:**

Our updated meta-analysis showed that aspirin was inferior to when compared with other anticoagulants in VTE-related orthopedic major surgery, including proximal DVT and/or PE, and was more likely to form VTE. No differences between groups were identified for bleeding, wound complications, wound infections, transfusion, or death events.

## 1. Introduction

For patients undergoing hip and knee replacement, pelvic, and lower extremity trauma fractures surgery, the incidence of postoperative lower extremity venous thromboembolism (VTE) and pulmonary embolism (PE) is high; VTE incidences can be as high as 41% and proximal deep vein thrombosis (DVT) incidences up to 10.2%.^[1,2]^ VTE is a serious cause of unintended death in patients in perioperative periods, while fatal PE can occur in acute phases. Also, subacute/chronic formation of post-thrombotic syndrome, PE or chronic thromboembolic pulmonary hypertension, and high VTE recurrence rates can reduce quality of life or generate fatal long-term sequelae.^[3]^

VTE prevention mechanisms include: basic, physical, and pharmacological prophylaxis. Except for contraindications, pharmacological prophylaxis is included in all standard prophylaxis protocols to prevent VTE, and standardized pharmacological prophylaxis anticoagulation is used to reduce VTE complications. In recent years, different guidelines have been published on preventing and treating VTE using aspirin; The American Society of Hematology does not recommend aspirin as an anticoagulant prophylaxis and treatment,^[4]^ the National Institute for Health and Care Excellence^[5]^ does not recommend aspirin as a stand-alone anticoagulant for total hip arthroplasty (THA), total knee arthroplasty (TKA), and hip fracture, and The American Association for the Surgery of Trauma/American College of Surgeons-Committee^[6]^ does not recommend aspirin as an anticoagulation option after trauma. In American College of Chest Physicians guidelines^[7]^ (2022 edition), aspirin can be used as a stand-alone anticoagulant after THA/TKA/hip fracture, but low molecular heparin is the preferred choice. Thus, different guidelines espouse different views on aspirin for preventing lower extremity VTE in major orthopedic surgery.

Previously, aspirin has demonstrated high safety profiles due to its convenience and cost effectiveness; a large randomized placebo-controlled DVT prevention study after 13,356 hip fractures and 4088 knee replacements showed that aspirin reduced PE risk and prevented DVT after major orthopedic surgery, thereby confirming that aspirin had potential as a routine prophylactic agent for VTE formation in clinical practice.^[8]^ The role of aspirin in preventing VTE in major orthopedic surgery was previously reported in a 2022 meta-analysis,^[9]^ but the emergence of new large-scale randomized controlled trials (RCTs) and differences across studies have led to data equivocation.^[10,11]^ Therefore, we reanalyzed the efficacy and safety of aspirin when compared with other anticoagulants in preventing VTE in major orthopedic surgery.

## 2. Methods

This evidence-based analysis was performed according to 2020 Preferred Reporting Items for Systematic Reviews and Meta-Analyses guidelines^[12]^ and prospectively registered in PROSPERO (CRD42023407713). Using PubMed, The Cochrane Library, Embase, and Web of Science databases, a RCT literature search was conducted in August 2023. We searched databases using the following terms: aspirin, orthopedic surgery OR operation OR perioperation OR arthroplasty, venous thromboembolism, and randomized controlled trial. Additionally, using predesigned questionnaires, 2 authors independently extracted data and disagreements were resolved by another senior researcher.

Using the patient, intervention, comparative, study design and outcomes protocol to develop this review, our study questions were: the VTE incidence after major orthopedic surgery, including hip and knee replacement and trauma surgery (P) is high, and the VTE incidence is significantly reduced after routine postoperative anticoagulation. Aspirin has been favored by patients and physicians in the last 2 years, but its anticoagulation is controversial compared to other anticoagulants (C), but in RCTs (S) who has a better outcome in VTE, bleeding, wound complications, wound infection, transfusion, or death events achieve better outcomes (O)?

### 2.1. Inclusion and exclusion criteria

All literature comparing aspirin with other anticoagulant outcomes in major orthopedic surgery were included. Outcomes were: VTE, bleeding events, wound complications, wound infections, blood transfusions, and death events. Inclusion criteria were comparative studies on the use of aspirin and other anticoagulants for thromboprophylaxis in major orthopedic surgery. Exclusion criteria were: (1) nonhuman studies; (2) non-randomized clinical trials; and (3) studies not including aspirin. Excluded were reviews, letters, case reports, meeting abstracts, studies where aspirin was not compared with other anticoagulants, or studies where 2 other anticoagulants were combined and compared with aspirin prophylaxis, or studies only examining physical aspirin prophylaxis.

### 2.2. Quality assessments

Data extraction, analysis, and literature quality assessments were independently performed by 2 investigators, with disagreements resolved by reassessment between both investigators and discussion with a third to reach a final decision. Literature quality and the risk of bias were assessed using the Cochrane Collaboration Network Evaluation Risk of Bias tool as recommended by the Cochrane Handbook 6.3 (https://training.cochrane.org/handbook/current).^[13]^ The following 7 areas were evaluated: random assignment sequences, allocation scheme concealment of participant-subject blinding, blinding for outcome assessment, incomplete outcome data, selective reporting of results, and other methodological quality assessment biases. The following data were extracted from selected studies: first author, publication year, sample size, age, mechanical VTE prophylaxis, VTE diagnosis method, follow-up duration, treatment modality and duration, and clinical outcomes. This latter category included VTE as a main outcome. Additional outcomes included bleeding events, wound complications, wound infections, blood transfusion, and death events.

## 3. Statistical analysis

Statistical analyses were performed in Review Manager 5.3 (Cochrane Collaboration, Oxford, UK). Risk ratios (RR) with 95% confidence intervals (CI) were used to compare binary variables. For meta-analyses, the Cochrane Q *P* value and l^2^ statistic were used to check heterogeneity. When a *P* value < 0.1 or l^2^ > 50%, significant heterogeneity was indicated and a random-effects model was used to merge results. Otherwise, a fixed-effects model was used. A *P* value <.05 was considered statistically significant. We used Forest and funnel plots to identify publication bias. Subgroup analyses were conducted to explore sources of heterogeneity.

## 4. Results

**Figure 1.**
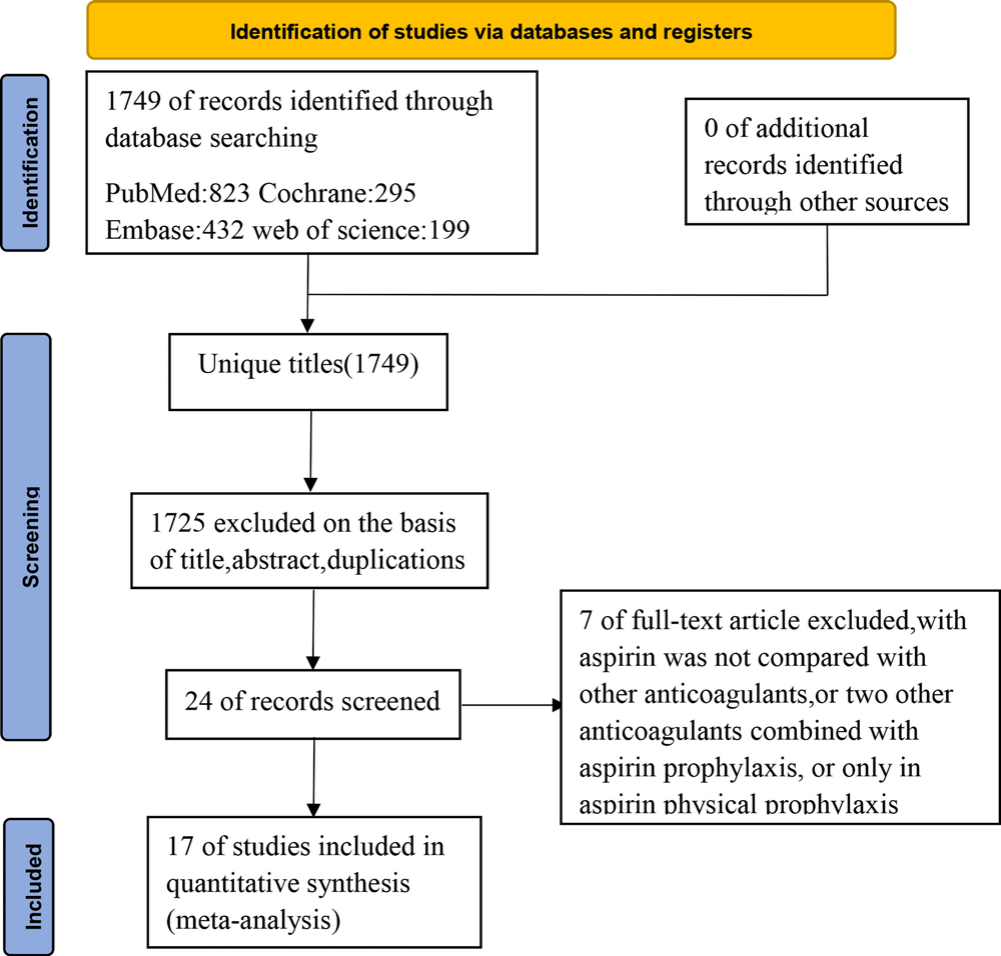
Study flowchart.

The systematic search and selection process is shown (Fig. 1). In total, 1749 relevant articles were identified in PubMed (n = 823), The Cochrane Library (n = 295), Embase (n = 432), and Web of Science (n = 199) using our systematic search strategy. After reading titles, abstracts, and removing duplicates, 1725 articles were excluded; 7 were further excluded because aspirin was not compared with other anticoagulants. Therefore, 17 eligible articles including 29,522 patients (15,253 on aspirin versus 14,269 on other anticoagulants) were analyzed.

**Figure 2.**
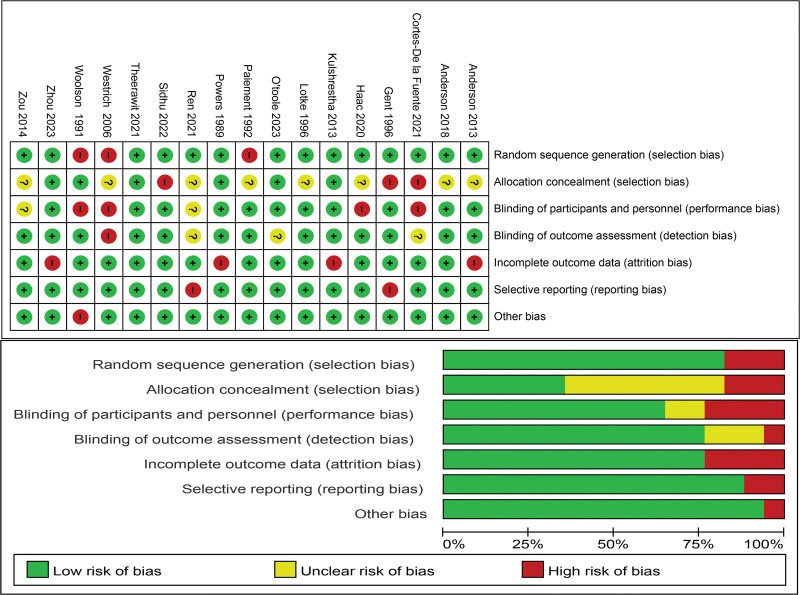
Study quality assessments using the Cochrane Risk of Bias Manual.

Selected studies were evaluated using RevMan 5.3 software according to the Cochrane Handbook of Systematic Evaluation Bias Assessment Manual (Fig. 2).

Study characteristics are shown (Table 1). In total, 29,522 patients were enrolled in the meta-analysis (15,253 aspirin versus 14,269 other anticoagulants). Studies were published between 1989 and 2023, and 13 and 4 arthroplasty and trauma surgery studies were included, respectively.

**Table 1:** Clinical characteristics of included studies.

Author	Year	No. of participants	Mean age, years			Gender (M:F)		VTE diagnostic methods	Follow-up duration	Physical prevention of VTE	Treatment dose and duration		Outcomes
			Aspirin	Other anticoagulants		Aspirin	Other anticoagulants				Aspirin	Other anticoagulants	
Powers	1989	194trauma	73.0	74.5		19:47	23:42	Iodine 125-fibrinogen leg scanning and Impedance plethysmography and/or venography	90 d	Not mentioned	66 cases 650mg twice daily on the first day after surgery	65 cases of warfarin 10 mg given as soon as possible after surgery	Proximal VTE or PE occurred in 6 patients in the warfarin group (9.2%), 7 patients in the aspirin group (10.6%), and 19 patients in the placebo group (30.2%) and was statistically highly significant (*P* = .005); warfarin was significantly more effective than aspirin or placebo, with little difference between aspirin and placebo; bleeding and death in all 3 groups were not statistically significant.
Woolson	1991	217THA	62.3	67.6		36:36	31:38	Venous ultrasonography and/or venography	>90 d	Intermittent inflation and compression device, elastic stockings	72 cases received oral 650 mg twice daily starting the night before surgery	69 cases oral warfarin 7.5 or 10 mg, adjusted according to prothrombin time	Proximal DVT: intermittent inflatable compression device + aspirin and intermittent inflatable compression device + warfarin occurred in 7 (10%) and 6 (9%) cases, respectively (*P* = .8), and 1 PE occurred in the aspirin group; each had 1 wound hematoma.
Paiement	1992	855THA	>39	>39		167:190	125:129	Venography	8–12d after surgery	Elastic stockings, balance suspension	43 cases 300 mg 224 case 1200 mg 90 cases 3600 mg	85 cases of conventional dose 10mg warfarin, 193 cases of low dose 5 mg warfarin	DVT: conventional dose of warfarin and low dose of warfarin provided statistically significant protection against DVT(*P* < .005 and *P* < .0001); Bleeding complications:1 case aspirin (0.45%)vs13 cases warfarin (15.3%) vs 3 cases low dose warfarin (1.6%)
Lotke	1996	133THA or 179TKA	66.4	67.1		66:100	55:91	Ventilation perfusion scan and venography	6 mo	Physical prevention	62 THA, 104 TKA 1 tablet (325 mg) twice daily, starting on the day of admission	71THA,75TKA Warfarin dose of 10 mg on the night of surgery, dose adjusted according to prothrombin time, switch to aspirin prophylaxis after 6 weeks postoperatively	VTE: there was no difference in the incidence between the aspirin and rivaroxaban groups (*P* > .05).Bleeding was not statistically significant in the warfarin and aspirin-treated groups. Wound complications:7 patients warfarin-treated had prolonged wound drainage requiring braking vs 6 patients in the aspirin-treated. After 48 hours postoperatively, no patient required blood transfusion due to bleeding.
Gent	1996	251trauma	76.6	76.6		24:102	23:102	Iodine 125-fibrinogen leg scanning and impedance plethysmography and/or venography	14 d	Not mentioned	126 cases 100 mg twice daily, starting 12–24 h postoperatively	125 cases low molecular heparin 750 units q 12 h, starting 12–24 h postoperatively	DVT:25 patients (27.8%) with low molecular heparin and 39 patients(44.3%) with aspirin(*P* = .028), proximal DVT or PE: 6 patients (6.8%) with low molecular heparin and 12 patients (14.3%) with aspirin (*P* = .137), bleeding complications: 2 (1.6%) in the low molecular heparin group and Aspirin 8 (6.4%) (*P* = .10).
Westrich	2006	275TKA	69.0	68.9		53:83	46:93	Venous ultrasonography	4–6 wk after surgery	Intermittent inflation and compression device	129 cases postoperative day 325 mg, twice daily, for 4 wk	135 cases Enoxaparin 30 mg bid was started 48 hours after surgery, and enoxaparin 40 mg qd was discharged for 3 weeks after anticoagulation	DVT:17 patients (12.6%) in 129 aspirin groups vs 18 patients (14.0%) in the 135 enoxaparin (*P* = .34), 1 PE in the aspirin group. Proximal DVT: 5 patients in the enoxaparin group and 2 patients in the aspirin group (*P* = .23). Bleeding complications: 1 intra-organ bleeding complication in the aspirin group.
Anderson	2013	778THA	57.6	57.9		231:154	213:18	Venography	90 d	Not mentioned	386 cases of THA on the second day after surgery up to 5000 U of dalteparin for 10 days and 81 mg of aspirin for 18 days	400 cases of THA up to dalteparin lasting 28 days	VTE: 5 (1.3%) patients dalteparin vs 1 (0.3%)patients aspirin (*P* = .22). Bleeding: 0 patients aspirin vs 1 patients dalteparin; 4 patients(1.0%) of incisional hematoma in the dalteparin vs 2 (0.5%) patients aspirin; 18 patients (4.5%) of minor bleeding in the dalteparin vs 8 patients (2.1%) in the aspirin (*P* = .164).Wound complications:10 patients (2.5%) dalteparin vs 12 patients (3.1%) aspirin (*P* = .67).
Kulshrestha	2013	900TKA	Not mentioned			Not mentioned		Venous ultrasonography	6 wk, 3 mo, 6 mo and 1 year	Foot pump, intermittent inflation and pressure device	450 anticoagulation in the risk group *P*: score < 2, once 325 mg q12h for 4 weeks starting from the first postoperative day	450 conventional anticoagulation group: 20mg of enoxaparin subcutaneously 8h postoperatively, 40mg on the first day postoperatively, for 2 weeks, and aspirin for 2 weeks at discharge. score > 2 two weeks of low molecular heparin anticoagulation followed by aspirin for 2 weeks (325 mg twice daily)	VTE: 15 (2.1%) patients enoxaparin vs 4 (2.1%) patients aspirin (*P* = .957). Wound complications:56 (7.9%) patients enoxaparin vs 2 (1.0%) patients aspires (*P* = .0005).
Zou	2014	324TKA	62.7		63.5 65.7	28:82	20:92 32:70	Venous ultrasonography	4 wk	Not mentioned	110 cases 100mg/d, for 14 d	102 cases of rivaroxaban 10mg/day, 112 cases of subcutaneous injection of low molecular heparin 4000AxaIU(0.4 mL)/day，for 14 d	DVT:3 (2.94%) patients rivaroxaban vs 18 (16.36%) patients aspirin(*P* = .017), but rivaroxaban increased postoperative blood loss and wound complications in patients (*P* < .05).
Anderson	2018	3424 (1804THA, 1620TKA)	62.9 ± 10.1		62.7 ± 10.1	804:903	833:884	Ventilation-perfusion lung scan or computed tomography pulmonary arteriography; venous ultrasonography	90 d	Intermittent inflation compression device Gradient compression stockings, both or neither	Rivaroxaban 10 mg qd starting 6 hours or day 1 postoperatively until day 5 postoperatively; TKA and THA were anticoagulated with oral aspirin 81 mg for 9 days and 30 days, respectively	Rivaroxaban 10 mg TKA for 14 days, THA for 35 days	VTE:11 (0.64%) patients aspirin vs 12 (0.70%) patients rivaroxaban (*P* = .84). Complications of major bleeding occurred in 8 (0.47%) patients aspirin vs 5 (0.29%) patients rivaroxaban (*P* = .42). Clinically significant bleeding occurred in 22 (1.29%) patients aspirin vs 17 (0.99%) patients rivaroxaban (*P* = .43).
Haac	2020	329trauma	48.0		45.4	104:61	119:45	Ventilation-perfusion lung scan or computed tomography pulmonary arteriography; venous ultrasonography	90 d	Intermittent inflation compression device	165 cases trauma 81 mg bid, duration of anticoagulation not specified, 28 cases only during hospitalization,4 cases more than 28 days prophylaxis	164 cases enoxaparin 30 mg bid, duration of anticoagulation not specified, 57 cases only during hospitalization, 3 cases more than 28 days prophylaxis	Complications:99 (59.9%) patients aspirin vs 98 (59.4%) patients enoxaparin (*P* > .05).The numbers of VTE, bleeding, infection, and death events in the aspirin (enoxaparin) were 10 (10 cases), 42 (41 cases), 12 (14 cases), and 1 (2 cases), respectively.
Cortes-De la Fuente	2021	402TKA	70.33		71.31	102:87	106:108	Not mentioned	90 d	Not mentioned	188 cases 100 mg postoperative day 1 to 30 days	214 cases enoxaparin 40–60 mg postoperative day 1 to 30 days	DVT: 3 patients enoxaparin vs 2 patients aspirin (*P* < .23). Bleeding: 4 patients enoxaparin and 3 patients aspirin (*P* = .82), 5 patients of enoxaparin (1.24%) superficial surgical wound infection and 1 case of acute myocardial infarction.
Hongnaparak	2021	40TKA	68.15 ± 7.43		70.5 ± 7.25	7:13	3:17	Venous ultrasonography	14 d	Not mentioned	20 cases of 300 mg, duration of anticoagulation not specified	20 cases of rivaroxaban 10 mg, duration of anticoagulation not specified	Subcutaneous petechiae: 3 patients aspirin vs 5 patients rivaroxaban (*P* = .292)
Ren	2021	70THA	54.5 (40.8–62.3)		50.0 (36.8–57.0)	13:21	11:25	Venous ultrasonography	30 d	Intermittent inflation compression device	34 cases 100mg, twice daily, for 5 wk	36 cases rivaroxaban 10 mg, qd)	DVT: 8.8% patients aspirin vs 8.3% patients rivaroxaban (*P* = .91)
Sidhu	2022	9711THA or TKA	67		68	2467:3208	1733:2303	Not mentioned	90 d	Intermittent inflation compression device; Gradient compression stockings depending on the situation	5675 cases 100 mg qd orally 24 hours after surgery, THA for 35 days and TKA for 14 days	4036 cases of enoxaparin group 40 mg THA for 35 days and TKA for 14 days	VTE:187 (3.5%) patients aspirin and 69 (1.8%) enoxaparin (*P* = .007), with aspirin resulting in a significantly higher incidence of symptomatic VTE within 90 days. Secondary outcomes: mortality, major bleeding, readmission, reoperation at 90 days, reoperation at 6 months, no difference in medication adherence.
O’toole	2023	12211trauma	44.5 ± 18.0		44.7 ± 17.6	3832:2269	3769:2341	Not mentioned	90 d	Not mentioned	81 mg twice daily, for 21d	Low molecular heparin 30 mg twice daily, for 21 d	Deaths: 47 (0.78%) aspirin vs 45 (0.73%) low-molecular heparin (*P* < .001).DVT: 2.51% aspirin vs 1.71% low-molecular heparin.
Zhou	2023	180TKA	66.4 ± 7.6		64.8 ± 7.2 64.1 ± 6.7	26:34	27:33 29:31	Computed tomography pulmonary arteriography and or Venous ultrasonography	90 d	Not mentioned	60 cases 100mg orally 12 h postoperatively, for 30 d	60 cases of rivaroxaban group 10 mg or 60 cases of dalteparin sodium 2500IU 12h postoperatively, for 30 d	There was no significant difference between VTE, bleeding and blood transfusion in the aspirin group and the other 2 groups.

DVT = deep vein thrombosis; PE = pulmonary embolism; THA = total knee arthroplasty; TKA = total knee arthroplasty; VTE = venous thromboembolism.

**Figure 3.**
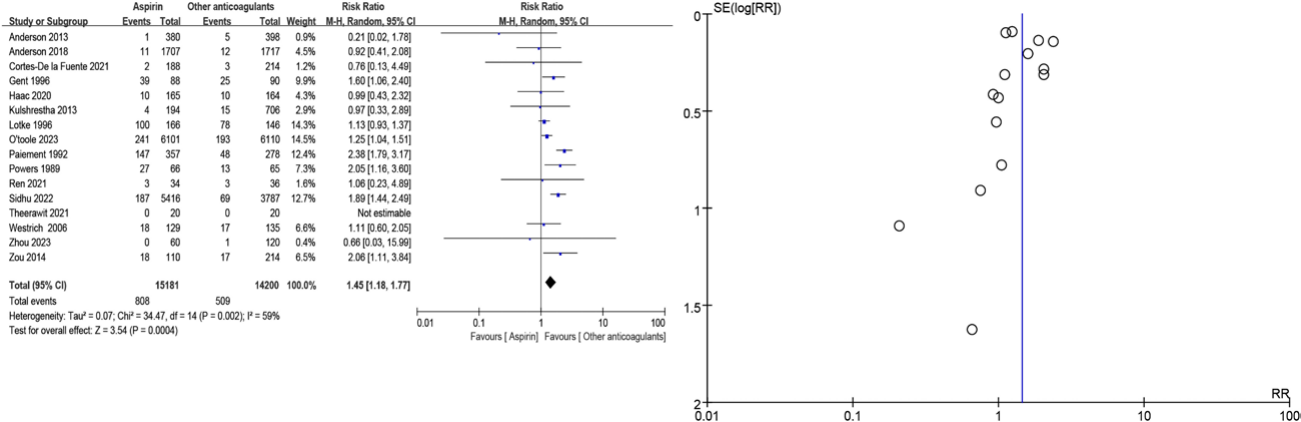
Venous thromboembolism forest and funnel plots.

**Figure 4.**
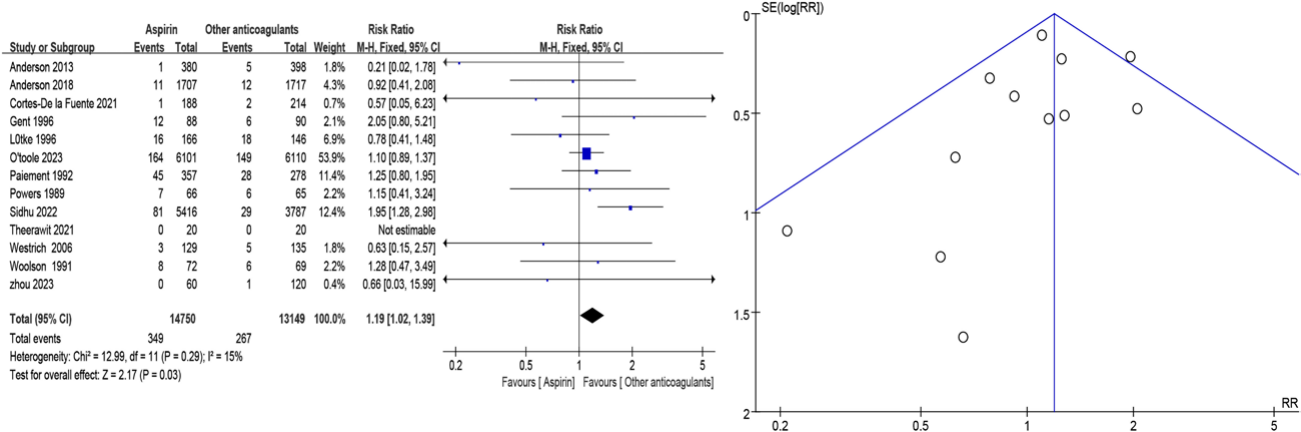
Proximal deep vein thrombosis/pulmonary embolism forest and funnel plots.

The 16 selected studies^[10,11,14–27]^ assessed DVT in major orthopedic surgery: the VTE incidence was more high in the aspirin group when compared with other anticoagulants (RR = 1.45, 95% CI = 1.18–1.77, *P* = .0004). As high heterogeneity (*P* = .002, *I*^2^ = 59%) was identified (Fig. 3). Proximal DVT was defined as at least 1 DVT in the iliac, femoral, or popliteal vein in any one lower extremity: 13 studies^[10,11,14–17,19,21–23,25,26,28]^ assessed proximal DVT and/or PE incidence was more high in the aspirin group when compared with other anticoagulants (RR = 1.19, 95% CI = 1.02–1.39, *P* = .03). As low heterogeneity (*P* = .29, *I*^2^ = 15%) was identified (Fig. 4).

**Figure 5.**
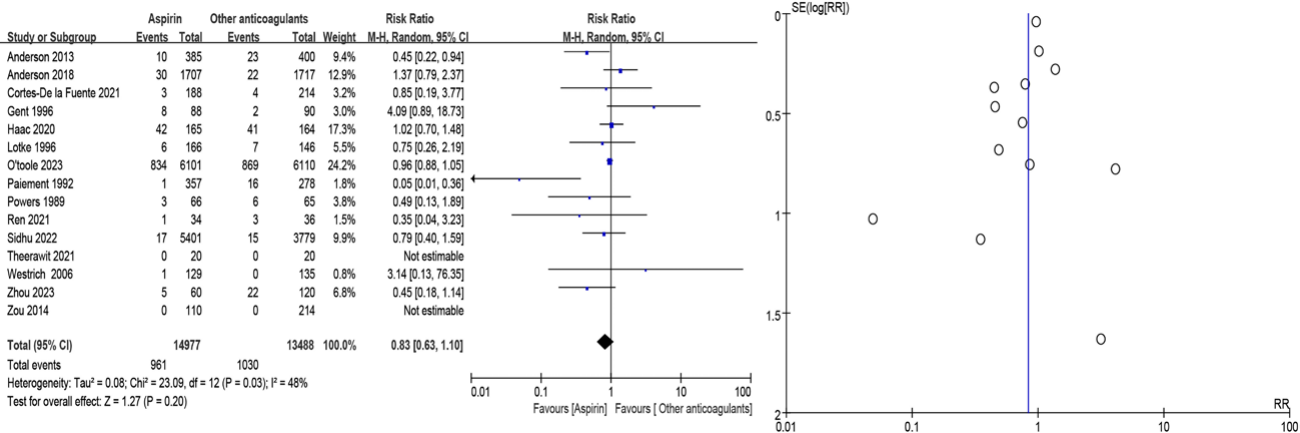
Bleeding event forest and funnel plots.

Bleeding events, including significant bleeding concomitant with reduced hemoglobin levels (>20 g/L), transfusion ≥ 2 units, organ bleeding, and abnormal bleeding at the surgical site, were included in 15 studies^[10,11,14–19,21–27]^ and showed no significant difference in the aspirin group when compared with other anticoagulants (RR = 0.83, 95% CI = 0.63–1.10, *P* = .20). As high heterogeneity (*P* = .03, *I*^2^ = 48%) was identified (Fig. 5).

**Figure 6.**
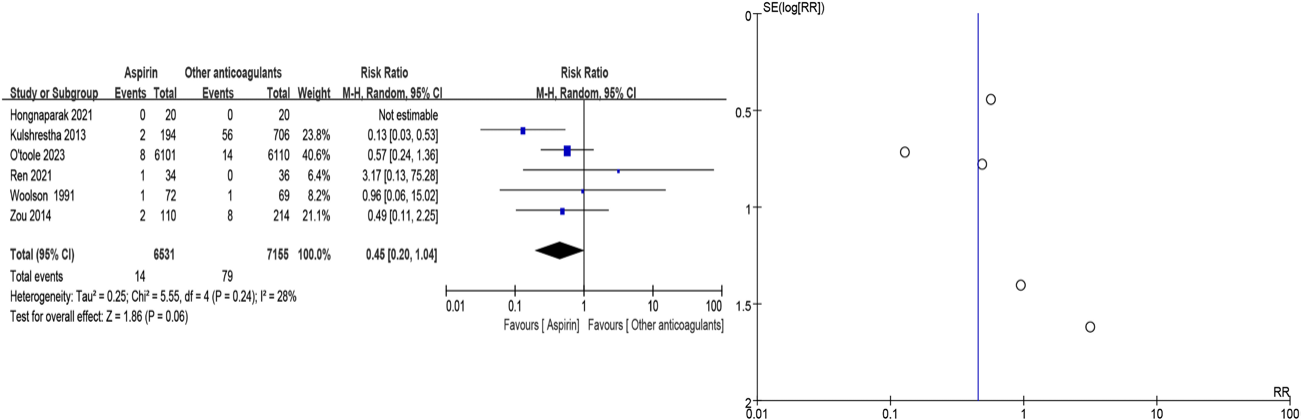
Wound complication forest and funnel plots.

Wound complications, including wound hematoma, persistent wound exudate, and erythema, were included in 6 studies^[11,19,20,24,27,28]^ and showed no significant differences in the aspirin group when compared with other anticoagulants (RR = 0.45, 95% CI = 0.20–1.04, *P* = .06). As low heterogeneity (*P* = .24, *I*^2^ = 28%) was identified (Fig. 6).

**Figure 7.**
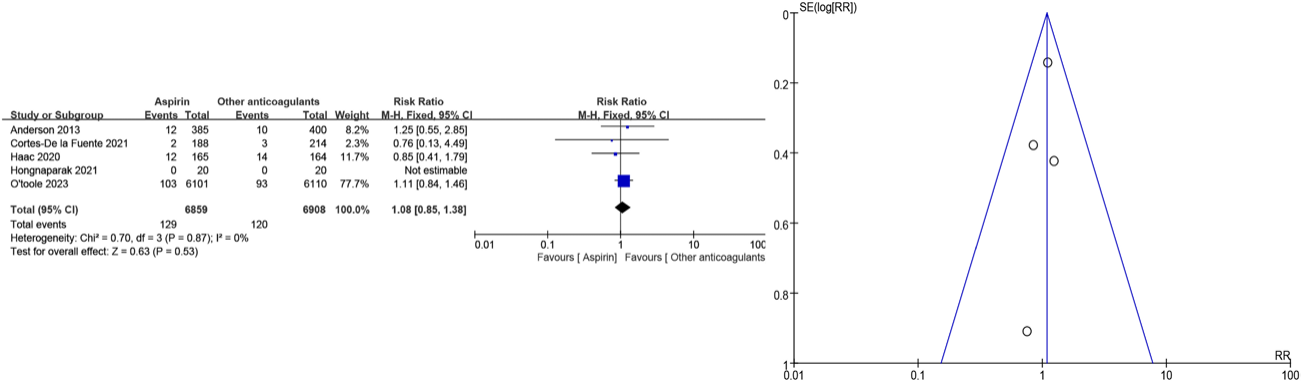
Wound infection forest and funnel plots.

Wound infections, including superficial wound infections and deep infections, were included in 5 studies^[11,14,16,18,19]^ and showed no significant differences in the aspirin group when compared with other anticoagulants (RR = 1.08, 95% CI = 0.85–1.38, *P* = .53). As low heterogeneity (*P* = .87, *I*^2^ = 0%) was identified (Fig. 7).

**Figure 8.**
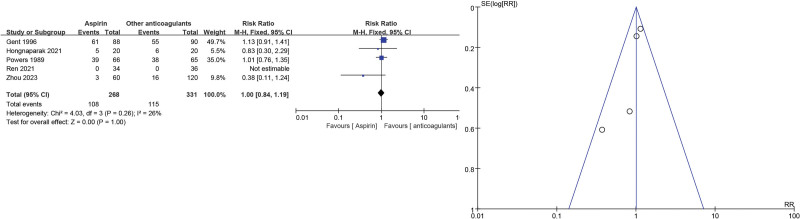
Transfusion event forest and funnel plots.

For transfusion events, 5 studies^[17,19,23,24,26]^ showed no significant differences in the aspirin group when compared with other anticoagulants (RR = 1.00, 95% CI = 0.84–1.19, *P* = 1.00). As low heterogeneity (*P* = .26, *I*^2^ = 26%) was identified (Fig. 8).

**Figure 9.**
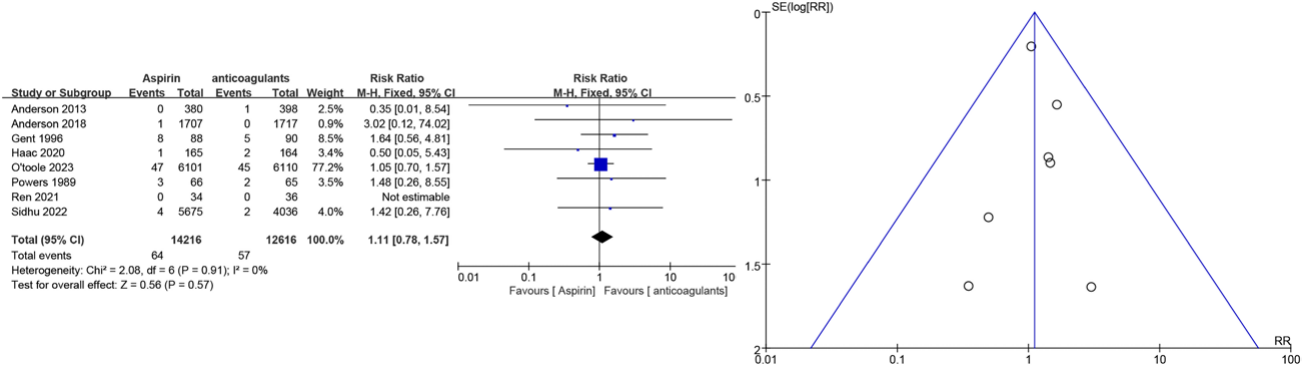
Death event forest and funnel plots.

For death events, 8 studies^[10,11,14,15,17,18,23,24]^ showed no significant differences between aspirin and other anticoagulants (RR = 1.11, 95% CI = 0.78–1.57, *P* = .55). As low heterogeneity (*P* = .91, *I*^2^ = 0%) was identified (Fig. 9).

**Figure 10.**
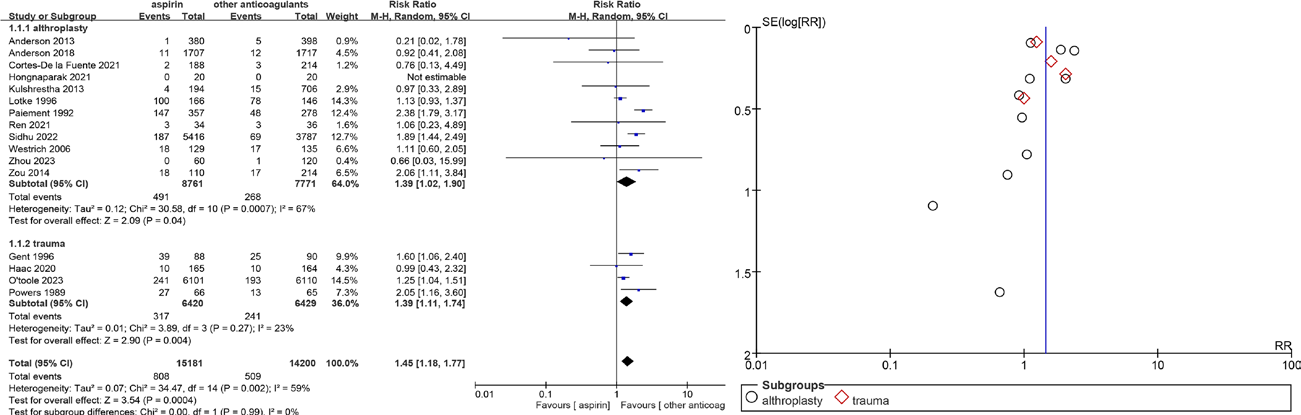
Trauma or arthroplasty surgery choice on venous thromboembolism forest and funnel plots.

Subgroup analyses also showed that aspirin was inferior to other anticoagulants in terms of VTE incidence in selecting the surgical approach for major orthopedic surgery (RR = 1.45, 95% CI = 1.18–1.77, *P* = .0004), with high heterogeneity (*P* = .002, *I*^2^ = 59%). Four trauma groups^11,17,18,23^ were included to assess the prevention of VTE in orthopedic trauma surgery, and the incidence of VTE in the aspirin group was inferior to that of other anticoagulants (RR = 1.39, 95% CI = 1.14–1.74, *P* = .004), *P* = .27, *I*^2^ = 23%), and 12 post-THA/TKA^10,14–16,19,21,22,24–27^ assessments were included for prevention of VTE, and the incidence of VTE in the aspirin group was inferior to that of other anticoagulants (RR = 1.39, 95% CI = 1.02–1.90, *P* = .04) with high heterogeneity (*P* = .0007, *I*^2^ = 67%) (Fig. 10).

**Figure 11.**
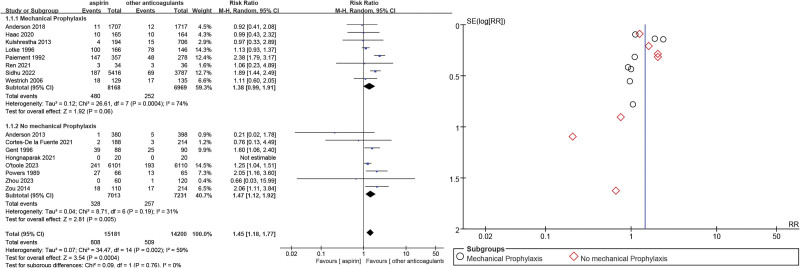
Combined with mechanical prevention of venous thromboembolism forest and funnel plots.

We performed subgroup analyses also showing that aspirin was inferior to other anticoagulants for the prevention of VTE incidence in major orthopedic surgery (RR = 1.45, 95% CI = 1.18–1.77, *P* = .0004), with a high degree of heterogeneity (*P* = .002, *I*^2^ = 59%). Eight studies^10,15,18,20–22,24,25^ examined combined anticoagulants and physical prophylaxis for VTE, and aspirin combined with mechanical prophylaxis resulted in a non-inferior incidence of VTE to the other anticoagulants (RR = 1.38, 95% CI = 0.99–1.91, *P* = .06) with high heterogeneity (*P* = .0004, *I*^2^ = 74%). Eight studies of anticoagulants (not combined with mechanical VTE prevention)^11,14,16,17,19,23,26,27^ showed that the incidence of VTE associated with aspirin alone was inferior compared to other anticoagulants (RR = 1.47, 95% CI = 1.12–1.92, *P* = .005) (Fig. 11).

## 5. Discussion

We found that aspirin was significantly more likely to generate VTE when compared with other anticoagulants in major orthopedic surgical procedures, including proximal DVT and/or PE. Subgroup analyses showed a significant difference between aspirin and other anticoagulants in preventing VTE in arthroplasty or trauma orthopedic surgery, with aspirin inferior to other anticoagulants in preventing VTE in either joint or trauma surgery. Significant differences were identified between aspirin and other anticoagulants in preventing VTE in major orthopedic surgery, with aspirin inferior to other anticoagulants in preventing overall VTE, but not inferior to other anticoagulants in preventing VTE in combination with machinery, and inferior to other anticoagulants in preventing VTE with aspirin alone. No differences between groups were identified for bleeding, wound complications, wound infections, transfusion, or death events.

Some anticoagulants used for major orthopedic surgery have significant drawbacks when compared with aspirin, and are not preferred by patients and physicians. Regular heparin, low-molecular weight heparin (LMWH), and sulfadoxine sodium require daily injections, while warfarin requires regular blood sampling to monitor international normalized ratios, but bleeding risks are high with excessive anticoagulation. Aspirin is an effective anticoagulant in preventing VTE and has low bleeding risks when compared with other anticoagulants due to its safety, ease of use, low price, and good patient compliance profiles, as indicated by a large combined database of 18,288 post-TKA anticoagulant choices.^[29]^ However, our findings suggested that clinicians should use caution when using aspirin as an anticoagulant in major orthopedic surgery. Previous meta-analyses comparing aspirin and other anticoagulant efficacy and safety profiles in preventing VTE after hip and knee replacement or orthopedic trauma reported no statistically significant differences in preventing DVT when compared with hip and knee replacement and hip fracture after surgery. Aspirin had the same effects, but overall quality assessments of selected RCTs ranged from low to high quality, and most RCT evidence showed a high risk of bias.^[9,30–32]^ Another meta-analysis reported no statistically significant differences between aspirin and enoxaparin for DVT and PE; overall evidence quality was low and validation is required from more RCTs.^[33]^ Coveney^[34]^ et al supported aspirin as effective in preventing symptomatic VTE after THA in 8885 patients receiving aspirin. However, we observed that LMWH anticoagulation during hospitalization and continued oral aspirin for 6 weeks after discharge was significantly intrusive. He et al^[35]^ investigated the effectiveness and safety of different anticoagulants in preventing VTE in hip and knee replacements using a reticulated Meta-analysis. Fondaparinux and rivaroxaban were the most effective anticoagulants in patients undergoing THA or TKA, and aspirin, although showing shown some anticoagulant effect, the anticoagulant effectiveness and safety of bleeding gives rise to cautious consideration, associated with the limitation of including only 2 randomized studies of aspirin. Additionally, other Meta-analyses indicated that aspirin had significantly higher DVT incidence rates when compared with other anticoagulants; it should only be used as part of a multimodal, accelerated rehabilitation program and be cautiously used during orthopedic surgery.^[36,37]^ Le et al^[36]^ concluded that rivaroxaban was more effective than aspirin in preventing DVT risks in their analysis of 8 high quality studies; aspirin was safer than rivaroxaban in lowering transfusion rates and had significantly higher DVT incidence rates and lower transfusion risk. Farey et al,^[37]^ in their Meta-analysis on aspirin and enoxaparin after hip and knee arthroplasty, reported that aspirin may be a safe alternative to enoxaparin in patients with TKA, but due to a lack of trial data recommending caution in patients with THA, their 4 RCTs exhibited low quality and insufficient evidence to estimate VTE incidences or mortality rates with certainty. Richardson et al^[38]^ examined a national database containing 30,813 post-TKA anticoagulant studies and showed that LMWH, factor Xa inhibitors, and sulforaphane reduced VTE risks by at least 50% when compared with aspirin, however, these drugs also had higher bleeding complication rates, while aspirin post-TKA anticoagulation showed high VTE incidences and low bleeding risks. Thus, anticoagulation strategies should be selected using balanced risk/benefit drug profiles to prevent VTE in patients. Runner et al^[39,40]^ investigated the American Board of Orthopedic Surgery database, which included 22,072 primary hip and knee arthroplasties and 6387 revision arthroplasties. Individual reasons for using VTE prophylaxis drug methods was unknown, but anticoagulants such as LMWH (enoxaparin), warfarin, rivaroxaban, and sulfonamide were associated with higher bleeding rates and thrombotic complications, whereas aspirin and/or mechanical device compression alone were not associated with higher thrombosis rates.

### 5.1. Study strengths and limitations

Strengths: (1) total sample size was large and we were the first meta-analysis to include 2 of the largest aspirin RCTs,^[10,11]^ thereby providing a comprehensive update of aspirin efficacy and safety when compared with other anticoagulants in preventing VTE after major orthopedic surgery. Our search strategy was comprehensive and detailed, involved multiple databases, had no language restrictions, included only RCTs, excluded observational studies, and removed inherent selection bias associated with our study design. (2) Subgroup analyses were performed in 2 ways, aspirin versus other anticoagulants for the prevention of VTE in different major surgical options in orthopedics and aspirin and other anticoagulants with or without combined mechanical prevention of VTE, to explore the sources of heterogeneity from multiple perspectives, and to fully explore the comparison of the effects of aspirin and other anticoagulants in the prevention of VTE.

Study limitations: (1) a population subgroup analysis could not be performed. There was no evidence of aspirin effectiveness and safety when compared with other anticoagulants in preventing VTE in high risk VTE patients, thus high-risk VTE prophylaxis may have been appropriately prescribed with LMWH anticoagulation, while favorable aspirin results may be attributable to patient selection. Mihara et al^[41]^ reported effective prophylactic use of low-dose aspirin in 3295 patients at high VTE risk after THA. However, patients received anticoagulants (enoxaparin or edoxaban) for 5 days after surgery and the authors could not demonstrate the effectiveness of aspirin alone in preventing VTE in high risk VTE patients. (2) VTE may include proximal DVT and/or PE; some studies may not have distinguished between these conditions and were thus uniformly classified as VTE. (3) There was no way to standardize aspirin doses; selected studies used a range of doses (81–3600 mg), with no uniform dosing criteria. Faour et al^[42,43]^ also showed that VTE prophylaxis was studied in THA/TKA cases in the low-dose aspirin group (81 mg twice daily) and standard-dose (325 mg twice daily), and the results showed that there was no significant difference in the incidence of symptomatic VTE, bleeding, and death after THA/TKA in the small-dose aspirin group as compared with standard-dose aspirin. Low-dose aspirin may be a safe and effective anticoagulant for the prevention of VTE after THA/TKA.

## 6. Conclusions

We showed that aspirin was inferior to for VTE when compared with other anticoagulants in orthopedic major surgery, including proximal DVT and/or PE, and was more likely to form DVT. No differences between groups were identified for bleeding, wound complications, wound infections, transfusion, or death events.

## Author contributions

**Conceptualization:** Heng-Zhi Liu, Jie Liang, Ai-Xin Hu.

**Data curation:** Heng-Zhi Liu.

**Formal analysis:** Heng-Zhi Liu, Ai-Xin Hu.

**Software:** Heng-Zhi Liu.

**Supervision:** Jie Liang, Ai-Xin Hu.

**Visualization:** Heng-Zhi Liu.

**Writing – original draft:** Heng-Zhi Liu, Jie Liang, Ai-Xin Hu.

**Writing – review & editing:** Heng-Zhi Liu, Jie Liang.
